# Neurophysiological Effects of Chronic Indoor Environmental Toxic Mold Exposure on Children

**DOI:** 10.1100/tsw.2003.22

**Published:** 2003-04-28

**Authors:** Ebere C. Anyanwu, Andrew W. Campbell, Aristo Vojdani

**Affiliations:** Cahers Neurosciences Research, 8787 Shenandoah Park Drive, Suite 122, Conroe, TX 77385, USA

**Keywords:** sick building syndrome, molds, mycosis, mycotoxins, neuropediatric effects, U.S.

## Abstract

The phenomenon of building-related diseases is attracting much research interest in recent years because of the extent to which it affects people with compromised immune systems, especially children. In this study, we reported the neurological findings in children who attended our Center because of chronic exposure to toxic molds. Clinical neurological and neurobehavioral questionnaires were administered with the cooperation of the children's parents. The children then underwent a series of neurophysiological tests including electroencephalogram (EEG), brainstem evoked potential (BAEP), visual evoked potential (VEP), and somatosensory evoked potential (SSEP). The results showed high levels of abnormalities in the analysis of the subjective responses derived from the questionnaires. The EEG examination was abnormal in seven out of ten of the patients compared to the controls with only one in ten with episodes of bihemispheric sharp activity. In all the patients, there was frontotemporal theta wave ativity that seemed to indicate diffuse changes characteristic of metabolic encephalopathies. Also, there was highly marked 1 to 3 Hz delta activity that was asymmetrical in the right hemisphere of the brain in three out of ten patients. The waveforms of BAEP showed abnormalities in 90% of the patients with both 15’ and 31’ check sizes compared to none in the controls. There were significant delays in waveform V in a majority of the patients representing dysfunctional cognitive process and conductive hearing loss in both ears. VEP showed clear abnormalities in four in ten of the patients with P100 amplitudes and latencies decreased bilaterally. In all the patients, there was slowing of conduction in the right tibial at an average of 36.9 ms and there was significant decrease in amplitude of response at the proximal stimulation site. Sensory latencies obtained in the median, ulnar, and sural nerves bilaterally showed abnormalities in five out of ten compared to none in the controls. The median, ulnar, and sural sensory potentials were abnormal in six out of ten patients. There was prolongation of the median distal sensory latencies bilaterally at an average of 4.55 ms on the right and an average of 6.10 ms on the left as compared to the ulnars of 2.55 ms bilaterally. There was no abnormality in the controls. These findings represent evidence of diffuse polyneuropathy to which three patients demonstrated borderline slow motor conduction at an average of 41.1 ms. Overall, the objective neurophysiological measurements (EEG, BAEP, VEP, and SSEP) were abnormal, indicating significant neurological deficits in all the patients. Our findings revealed the extent to which toxic molds can affect the neurological and behavioral status of children. Further work should be encouraged in this regard.

## INTRODUCTION

Building-related diseases are defined by the manifestations of symptomatic illnesses due to poor indoor environmental air quality and pollutant exposures. According to the WHO definition, it is characterized by irritations of mucosa, vegetative complaints such as headache and fatigue, as well as mental health impairment, depression, and irritability[1,2].

It is also defined as an increased occurrence of nonspecific symptoms among populations in determined buildings and this definition differs from those of other medical syndromes in that it refers to a system (a building inclusive of its population) rather than to a clinical state in a single individual[3,5]. Such a definition has made the term “sick building syndrome” (SBS) impossible to use as a diagnosis applied to individual persons[3,4,5]. Hence, we prefer to call this phenomenon “indoor environmental related diseases (IERD)”. The syndrome has apparently become one of the common health concerns in the world, especially in the developed countries where industrially related environmental pollutions favor the growth of opportunistic toxic molds.

## DISTRIBUTION OF TOXIC MOLDS

In recent years, climatic changes have dramatically ensued from extreme cold to extreme heat, from sporadic lack of rainfall to geographically devastating floods. All these conditions favor the rapid growth of molds, some of which are saprophytically opportunistic and toxic to humans. Although the adverse health effects of toxic molds on humans are still emerging and not fully understood, some of the etiological factors may include extreme conditions of the indoor environmental conditions such as dampness, moldy air, temperature, and humidity. Visible condensation on windows, high air humidity in the bathroom, moldy odor, and water leakage of the residential homes, are the sources of toxic mold–related illnesses and all indicators of dampness are related to an increase in all types of symptoms, significant even when adjusted for age, gender, population density, type of ventilation system, and ownership of the building[6]. In fact, both toxic and nontoxic molds are everywhere and under favorable conditions, grow luxuriantly and cause diseases. Hence, more and more people throughout the country are having mold-related medical problems. Homes are being bulldozed in Houston, schools are closing in Arizona, and buildings are being shut down in Florida due to toxic mold.

## SOURCES OF INDOOR TOXIC MOLD EXPOSURE IN CHILDREN

Sources of indoor toxic mold exposures in children include contaminated schools, home environments, and cereals. When indoor environmental conditions are rife, the toxic mold release their spores and volatile organic chemicals such as methanol, ethanol, methane, and other toxic compounds into the air. People inside the schools or homes breathe in the polluted air. Children are the most vulnerable. The children readily inhale the polluted air and become ill especially if their immune systems are not yet competent to handle the antigenic effects of toxicity. Several cases of this nature have been reported globally. [7,8]

In the U.S., there had been concerns about health and indoor air quality (IAQ) in homes and schools in which toxic molds were isolated from swab samples of visible growth under wet carpets, on wet walls, or behind vinyl wall coverings and the associations with adverse health effects observed among the children exposed to such environments. Jarvis et al.[9,10] investigated a cluster of cases of pulmonary hemosiderosis in infants in Cleveland, OH and found that *Stachybotrys atra* and two isolates of a related toxigenic mold, *Memnoniella echinata*, were responsible for the adverse health effects on infants. *M. echinata* produces two cytotoxic trichothecene mycotoxins, trichodermol (1a) and trichodermin (1b), as well as several griseofulvins[9]. *Alternaria alternata*, *Aspergillus* spp., *Bipolaris spicifera*, *Curvularia lunata*, *Epicoccum nigrum*, and *Fusarium solani* were isolated repeatedly from groups of patients among 96 diagnosed patients with allergic fungal sinusitis[11]. *E. nigrum* was obtained consistently from four patients, one of whom yielded mycelial masses consistent in morphology with *E. nigrum*. Of the predominant toxic molds recovered from air samples from selected patients' residences, 15 included the same species isolated from the mucin of its inhabitants[11].

## CLINICAL MANIFESTATIONS OF TOXIC MOLDS

The adverse effects of toxic molds were first identified more than 60 years ago following an epidemic of stomatitis, rhinitis, conjunctivitis, pancytopenia, neurological disorders, and death in horses[12]. Since then, several toxic molds including *Stachybotrys, Fusarium*, and *Aspergillus* have been identified in patients and in their homes. Toxic molds are particularly important because they produce mycotoxins that are biologically active and can produce a variety of physiological and pathologic changes in humans, including modulation of inflammation and altered alveolar surfactant phospholipid concentrations[12].

In this study, all the toxic mold–exposed children continued to suffer from multiple illnesses. These included frequent symptomatic headaches, fatigue, earache, abdominal and joint pains, mood swing, persistent coughs, nosebleeds, hyperactivity, memory loss, and allergic reactions. Their previous surgical history was negative of mycosis and family history was also noncontributory with the exception of corresponding time frames for similar illnesses among family members residing in the same homes. According to their medical records, they were all in excellent health conditions before the toxic mold exposures that predicated frequent doctor’s visits with reports of evidence of flu-like illnesses and upper respiratory problems.

## AIM OF THE STUDY

The aim of this study therefore, was to assess the neurophysiological effects of chronic indoor environmental toxic mold exposures on children.

## MATERIALS AND METHODS

### The Study Population

The study population was made up of ten children aged between >18 months and =/>15 years old, six (60%) female and four (40%) male, and ten sex- and age-matched controls. A majority of the children came from families that bought their homes 2 to 8 years prior to this study. They moved into their homes before renovating them, then pulled off old wallpaper and carpets that were soaked with urine and stains. Some moved from homes that tested positive for molds and thus carried with them mold-contaminated furniture and household wares. Less than 30% of the children were either born in homes that were already exposed to toxic molds or lived in homes that were not previously known to be toxic mold–exposed but were later flooded, moist, damp, and humid. All the homes were extensively tested by certified environmental science laboratories and found positive for toxic mold contamination. The predominant toxic molds found were *Stachybotrys, Aspergillus, Cladosporium*, and *Penicillium*. The controls, on the other hand, had no known history of chronic toxic mold exposures either at home or school.

## Electrophysiological Tests

Carefully designed neurological questionnaires were first administered with the cooperation of the children's parents. Then, all the patients and controls underwent clinical neurophysiological tests including electroencephalogram (EEG), brainstem auditory evoked potential (BAEP), visual evoked potential (VEP), somatosensory evoked potential (SSEP), and nerve conduction velocity using Biologic CEEG Graph 4 Model 810 Version 5.71 with Biologic EP Explorer. All tests were carried out according to the standard methods. The EEG examination included hyperventilation and intermittent photic stimulation (IPS) using Biologic PS2 photostimulator at intensity 1363 cd/m$^2$. International electrode placement 10-20 system was used. The photostimulator was positioned at a distance of 18 in. (45.72 cm) from subjects' eyes at a visual angle of 25$^o$ and was given first with the eyes open for 5 s, then 5-s eye closure, and another 5 s with the eyes closed. Both the patients and the controls were asked to sit at a distance of 170 cm from the stimulus source with their eyes fixated on the red spot at the center of the monitor.

Brainstem auditory response testing was carried out by stimulating each ear independently with rarefaction clicks at a rate of 4 to 8 Hz. After taking proper consideration to the patients' comfort, the stimulus intensity was adjusted to 70 to 80 dB. Although, this sound range might seem relatively high, it was the range to which the patients were perceptive and comfortable. The recording electrodes were placed at the vertex (Cz of the International 10-20 System) and both earlobes. That is, the BAEPs were recorded between Cz and Ai, with a second channel, Cz-Ac. This was because this channel placement aided in the identification of waves IV and V, which may be fused in the channel 1 waveform. BAEPs were carried out within 0.3 ms on two repeats.

This was necessary to achieve a check size of 15' and 31'. Nerve conduction velocity was carried out using TECA Synergy Multimedia EMG with multisync color SVGA monitor and Delux stimulator probe (TECAsynergy, Oxford Instruments Medical, Inc.). VEP test was performed with corrective lenses if needed. However for SSEP, adequate pretest measures were taken to avoid dispensing too noxious stimuli. For the reliability and reproducibility of tests, repeats were performed.

## Data Analysis

Data generated from the questionnaire were analyzed using power formula analysis[13] to calculate the number required to detect a difference between the mean score of the patients (abnormal) and controls (normal) responses. Values from power analysis formula for n to detect a 20% difference was 0.05 for abnormal; 0.05 for normal. Power (P) was 95% and the constant (K) was 3.29. Neurological response differences in mean change were assessed using an analysis of variance (ANOVA) model. Paired *t*-test comparisons between outcomes in each were conducted using least squares means from the above model. Confidence intervals of 95% for effect-exposure differences were computed on the basis of the least squares means from the ANOVA model. For binary measures, such as the percentage of neurological abnormalities, comparisons were conducted using Chi-square test. Unadjusted *p* values are reported. To assess symptom severity at endpoint relative to an unaffected population, we analyzed scores on the primary outcome measure as *t* scores. All statistical analyses were performed using StatiBot 2000–2002 software[14,15,16].

## RESULTS

The subjective neurological findings based on the clinical questionnaire and case histories are presented in Table 1. With a threshold of 0.05, n = 2646, ny = 20, $Ch^2 = 692$, $p < 0.0001$. A low *p* value means that the result was statistically significant. Hence, the neurological manifestations found among the patients were related to chronic mold exposures compared to the controls. Fatigue, headache, dizziness, cough, and nosebleeds were the most frequent (100%) occurrences.

**TABLE 1**
**Subjective Neurologic Responses with Parental Cooperation**

| Neurological Responses | No. (%) (n = 2 x 10) | |
|---|---|---|
| | *Patient* | *Control* |
| Fatigue | 10(100) | 0(0) |
| Headache | 10(100) | 0(0) |
| Dizziness | 10(100) | 0(0) |
| Feeling sick (nausea) | 7(70) | 3(30) |
| Cough | 10(100) | 0(0) |
| Shortness of breath without physical exercise | 9(90) | 1(10) |
| Feeling of general exhaustion | 9(90) | 1(10) |
| Heart fluttering (palpitations) | 5(50) | 0(0) |
| Bright light | 9(90) | 3(30) |
| Burning eyes | 7(70) | 0(0) |
| Ringing in your ears | 5(50) | 0(0) |
| Ear discharges | 4(40) | 0(0) |
| Hearing loss | 6(60) | 0(0) |
| Problems with balance | 4(40) | 0(0) |
| Runny nose | 8(80) | 2(20) |
| Nosebleeds | 10(100) | 1(10) |
| Poor appetite | 6(60) | 0(0) |
| Abdominal pains | 4(40) | 1(10) |
| Weakness of you arms and legs | 9(90) | 0(0) |
| Numbness in the limbs | 9(90) | 0(0) |
| Tingling in arms or legs | 5(50) | 0(0) |

### The EEG Examination

The EEG examination was abnormal in seven out of ten of the patients compared to the controls with only one in ten with episodes of bihemispheric sharp activity. It was later found that this particular control subject had had seizures of unknown etiology in the past. There was frontotemporal theta wave activity that seemed to indicate diffuse changes characteristic of metabolic encephalopathies. There was scattered and slowing of the alpha rhythm with subsequent appearance of theta and delta activity. This wave activity was enhanced by hyperventilation. There was certainly undefined triphasic wave activity in a majority of the patients that consisted of a minor positive potential preceded and followed by another smaller negative waves. In three out of ten patients in particular, there was highly marked 1- to 3-Hz delta activity that was asymmetrical in the right hemisphere of the brain. Sharp synchronous bilateral theta activity of 4 Hz was also observed and there were no such wave activity in the controls.

## BAEP Examination

The waveforms of BAEP showed abnormalities in 90% of the patients with both 15’ and 31’ check sizes compared to none in the controls. There were significant delays in waveform V in a majority of the patients. This may represent dysfunctional cognitive process and conductive hearing loss in both ears since the waveform V is known to be very sensitive in both patients and in normal subjects[17]. Although the interpeak latencies were symmetrical and bilaterally well developed in all the patients, probably because of their age however, the amplitudes were diminished and the latencies were prolonged in a majority of the cases.

## VEP Examination

Visual evoked response testing was carried out stimulating each eye independently with pattern shift stimulus. Although the waveforms were well developed bilaterally in all the patients, the VEP showed clear abnormalities in four in ten of the patients compared to one in ten of the controls. Four in ten of the patients’ P100 amplitudes and latencies were decreased bilaterally. The mean full-field pattern reversal for P100 latency was OS 120.17:OD 119.19 ms in the patients compared to OS 93.79:OD 93.63 ms in the controls. The mean P100 amplitude was OS 6.82:OD 5.19 μV compared to OS 21.42:OD 20.18 μV for the controls.

On hemifield testing there was also a definite prolongation of the latencies bilaterally in all the patients during stimulation. These findings may represent evidence of bilateral optic nerve dysfunction. One of the patients (8 years old) had visual disturbances with a visual acuity of 20/30 in the right eye. It was not possible to verify the visual acuity of the left eye due to nystagmus in that eye. Note the waveforms were well developed with right eye stimulation and less developed with left eye stimulation. The P100 latency was markedly prolonged with left eye stimulation at 143.62 ms and the amplitude of response was decreased at 1.26 μV. The P100 latency and amplitudes were well within normal limits with the right eye stimulation. This is an evidence of left optic nerve dysfunction.

## SSEP Examination

SEEP was performed by stimulating each posterior tibial nerve independently while recording at popliteal fosse lumbar point, cervical point, and scalp. There was prolongation of lumbar point to the scalp interpeak latency with left posterior tibial stimulation in five in ten of the patients. There was also evidence of a central conduction delay from lumber point with left posterior stimulation. This could represent sensorimotor polyneuropathy. In all the patients, there was slowing of conduction in the right tibial at an average of 36.9 ms and there was significant decrease in amplitude of response at the proximal stimulation site.

Sensory latencies obtained in the median, ulnar, and sural nerves bilaterally showed abnormalities in five out of ten compared to none in the controls. There was mild prolongation of the left median distal sensory latency at 3.5 ms and both ulnar distal sensory latencies at 3.15 ms on the left and 3.10 ms on the right. The mean parametric median nerve somatosensory measurements in both the patients and the controls are presented in Table 2. There were significant differences between the readings from the patients and the controls ($p < 0.001$).

**TABLE 2**
**Mean Parametric Median Nerve Somatosensory Measurements in Both the Patients and the Healthy Control**

| PARAMETER | PATIENTS | | CONTROLS | | X | SD | MIN | MAX |
|---|---|---|---|---|---|---|---|---|
| | *Left* | *Right* | *Left* | *Right* | | | | |
| ***Absolute latency (peak in ms)*** | | | | | | | | |
| EP | 10.03 | 9.95 | 9.56 | 9.72 | 9.7 | 0.76 | 7.9 | 11.2 |
| N13 | 12.32 | 12.64 | 12.32 | 12.64 | 13.5 | 0.92 | 11.5 | 15.6 |
| N19 | 18.09 | 18.25 | 17.22 | 17.62 | 19.0 | 1.02 | 16.7 | 21.2 |
| P22 | 20.78 | 20.86 | 19.67 | 20.22 | 22.0 | 1.29 | 19.1 | 25.2 |
| ***Interwave latency (ms)*** | | | | | | | | |
| EP-N13 | 2.29 | 2.69 | 2.76 | 2.92 | 3.8 | 0.45 | 2.7 | 4.5 |
| EP-N19 | 8.06 | 8.30 | 7.66 | 7.90 | 9.3 | 0.53 | 7.8 | 10.4 |
| EP-P22 | 10.75 | 10.91 | 10.11 | 10.50 | 12.3 | 0.86 | 10.0 | 15.0 |
| N13-N19 | 5.77 | 5.61 | 4.90 | 4.98 | 5.5 | 0.42 | 4.7 | 6.8 |
| ***L/R latency difference (ms)*** | | | | | | | | |
| EP | 0.08 | | 0.16 | | 0.2 | 0.20 | 0.0 | 0.9 |
| EP-N13 | 0.40 | | 0.16 | | 0.2 | 0.17 | 0.0 | 0.6 |
| EP-N19 | 0.24 | | 0.24 | | 0.2 | 0.21 | 0.0 | 0.8 |
| EP-P22 | 0.16 | | 0.39 | | 0.3 | 0.24 | 0.0 | 1.1 |
| N13-N19 | 0.16 | | 0.08 | | 0.3 | 0.25 | 0.0 | 1.1 |
| ***Amplitude (μς)*** | | | | | | | | |
| EP | 2.55 | 1.67 | 1.88 | 0.78 | 3.0 | 1.86 | 0.5 | 8.6 |
| N13 | 2.52 | 2.20 | 0.99 | 9.67 | 2.3 | 0.87 | 0.8 | 4.4 |
| N19 | 2.62 | 1.63 | 0.54 | 1.51 | 1.0 | 0.56 | 0.1 | 2.7 |
| P22 | 1.79 | 1.97 | 1.12 | 1.55 | 2.2 | 1.10 | 0.5 | 5.5 |
| ***L/R amplitude difference (μς)*** | | | | | | | | |
| N19 | 0.99 | | 0.97 | | 41.7 | 33.14 | 0.0 | 144.4 |
| P22 | 0.18 | | 0.33 | | 25.7 | 21.23 | 0.0 | 80.4 |

Stimulation rate = 4.7 ms.

## Nerve Conduction

Nerve conduction velocity studies were carried out in both upper and lower extremities. The median, ulnar, peroneal, and tibial nerves were evaluated with regard to motor conductions bilaterally. There was prolongation of the median nerve distal motor latencies bilaterally on the right and on the left of the patients, markedly low readings on the right and left of the ulnars. The median, ulnar, and sural sensory potentials were abnormal in six out of ten patients. There was prolongation of the median distal sensory latencies bilaterally at an average of 4.55 ms on the right and an average of 6.10 ms on the left as compared to the ulnars of 2.55 ms bilaterally. There was no abnormality in the controls. These findings represent evidence of diffuse polyneuropathy to which three patients demonstrated borderline slow motor conduction at an average of 41.1 ms.

## DISCUSSION

Unlike household allergens typically found indoors, the allergens from toxic molds such as *Aspergillus, Stachybotrys, Cladiosporium*, etc., are conceivably ubiquitous in homes and in schools that are exposed to dampness and dusty air. Homes with poor indoor air quality (IAQ) are also found to be frequently susceptible to toxic surface fungal contamination. Such molds can grow on building materials such as wallpaper, drywall, and ceiling tiles, particularly after water damage has occurred[18]. It was found that condensation on windows, high air humidity in the bathroom, moldy odor, and water leakage exposed children to fungal infection[6] leading to the neurophysiological effects such as ocular, nasal, throat, dermal symptoms, cough, headache, and tiredness. The prevalence of pulmonary hemorrhage/hemosiderosis due to indoor toxic mold exposures among children still remains valid[19,20,21,22,23,24].

However, the fact that molds can produce lethal mycotoxins is apparently becoming well established[25,26]. Nevertheless, the neurophysiological effects of chronic toxic mold exposure in children are emerging. In our patients, there were frontotemporal theta wave activities that seemed to indicate diffuse changes characteristic of metabolic encephalopathies. There was scattered and slowing of the alpha rhythm with subsequent appearance of theta and delta activity that was enhanced by hyperventilation. There was certainly undefined triphasic wave activity in a majority of the patients that consisted of a minor positive potential preceded and followed by smaller negative waves. These triphasic wave activities were previously thought to be specific for hepathic encephalopathy but are now believed to be in fact metabolic encephalopathy[25,26]. Our findings suggest that children who are chronically exposed to toxic mold may manifest either hypo- or hyperglycemia, hypo- or hyperthyroidism, or myexedema because of the low voltage record in which alpha activity was preserved with intermix of theta. There was also the presence of 4 Hz paroxysmal bilaterally synchronous generalized focal theta activities probably suggesting probably lesions to cortex. Although most of the activity varied with age, the presence of delta rhythms in a majority of the children was probably indicative of a destructive lesion of the cortex such as demyelination, infarction, or abscess.

The waveforms V of BAEP were abnormally delayed in all the patients. When the latency of P100 is outside 95 to 99% of normal boundaries or when there is unusual prolongation or absence of N75 of P100, or when there is an absence of VEP such as we have found in our study, there is no doubt that mycosis has a new dimension in neurologic science. It could be interpreted that not only did the children have dysfunctional cognitive process and conductive hearing loss, but also had some lesional disturbances in occipito- or paratemporal regions of the brain function. In this case, visual evoked response gave serious indications of clear abnormalities with possible optic nerve dysfunction. Nerve conduction study showed abnormalities that were consistent with neurological damage.

## CONCLUSIONS

Toxic molds can affect the nervous system in children who are chronically exposed. Common neurological symptoms are headaches, fatigue, sleep disturbance, confusion, mood swings, nosebleeds, and cough. High levels of sensorimotor dysfunction and the related disorders in children means that toxic mold exposure should be regarded as a serious neurological health concern in children indeed. Children can develop agitation and poor performance in school, which is sometimes misdiagnosed as hyperactivity. There are neurological tests that can help determine what has been damaged in the body of the children due to toxic mold exposures and how severely. Overall, the results seem to indicate that children are neurophysiologically vulnerable, especially those under 10 years of age. Although, further work in this topic is ongoing, further research investigations at other centers are encouraged.

## ACKNOWLEDGMENTS

We thank Ms. Nina Immers for her management support and invaluable administrative input in the execution of this research.

## BIOSKETCH

**Ebere C. Anyanwu** is a Clinical Neurophysiologist and the Director of Cahers Neurosciences Research Inc., Houston. Dr. Anyanwu is also an Adjunct Professor of Anatomy and Physiology at the North Harris and Montgomery Colleges in Houston and the Woodlands, TX.

**Andrew W. Campbell** is an Expert Medical Scientist in Immunotoxicology and the Medical Director and Chief Executive of the Center for Immune, Environmental and Toxic Disorders, Houston, TX.

**Aristo Vojdani** is a Professor of Immunochemistry and the Chief Executive of the Immunosciences Laboratory, Inc., Beverly Hills, CA. Dr. Vojdani is also Board Certified in Medical Laboratory Technology.
